# Contra-Directional Expression of Serum Homocysteine and Uric Acid as Important Biomarkers of Multiple System Atrophy Severity: A Cross-Sectional Study

**DOI:** 10.3389/fncel.2015.00247

**Published:** 2015-07-06

**Authors:** Dan Chen, Xiaobo Wei, Jing Zou, Rui Wang, Xu Liu, Xiaofeng Xu, Jianjun Lu, Zhanhang Wang, Beisha Tang, Brian Wang, Kunlin Jin, Qing Wang

**Affiliations:** ^1^Department of Neurology, The Third Affiliated Hospital of Sun Yat-Sen University, Guangzhou, China; ^2^Department of Neurology, Guangdong 999 Brain Hospital, Guangzhou, China; ^3^The State Key Laboratory of Medical Genetics, Central South University, Changsha, China; ^4^Department of Pharmacology and Neuroscience, University of North Texas Health Science Center, Fort Worth, TX, USA

**Keywords:** multiple system atrophy, homocysteine, uric acid, cognition, inflammation

## Abstract

**Highlights:**

- Serum Hcy was higher in MSA patients when compared to healthy subjects, particularly in male patients.
- Serum UA was lower in MSA patients when compared healthy subjects, particularly in male patients.
- Serum Hcy levels were significantly positively correlated with the severity of MSA.
- The ROC curve for the combination of Hcy and UA showed potential diagnostic value in discriminating MSA from healthy subjects.

**Aim:**

There is evidence suggesting that inflammatory responses play a critical role in the pathogenesis of multiple system atrophy (MSA). Whether inflammatory mediators can be used as reliable biomarkers to detect the severity and progression of MSA remains largely unknown.

**Methods:**

We performed a cross-sectional study that included 47 patients with MSA and 50 healthy age-matched controls. Serum levels of homocysteine (Hcy), uric acid (UA), and C-reactive protein (CRP) were measured. These levels positively correlated with the severity of MSA, based on both motor and non-motor symptoms. Several scales were used to rate the severity of MSA, including the Unified multiple system atrophy rating scale, Parkinson’s disease sleep scale, Non-motor Symptoms Scale, the Schwab & England activities of daily living scale, Webster Scale, modified Hoehn and Yahr staging scale, and the Mini-Mental State Examination. Receiver operating characteristic (ROC) curves was applied to map the diagnostic accuracy of MSA against healthy subjects.

**Results:**

Compared with healthy subjects, we found that serum Hcy was higher, UA was lower, and CRP levels were unchanged in MSA patients. These findings were especially prominent in male patients. No significant differences of serum Hcy and UA were observed between patients of MSA and PD. Interestingly, there was a significant correlation between Hcy levels and MSA severity such as movement dysfunction, declined cognition, and cardiovascular symptoms. Additionally, the ROC curve for the combination of Hcy and UA (AUC 0.736) showed potential diagnostic value in discriminating MSA from healthy subjects.

**Conclusion:**

Our findings suggest that the inflammatory mediators Hcy and UA may play important roles in the pathogenesis of MSA. The measurement of serum Hcy and UA levels could then be a useful tool to accurately distinguish MSA from healthy subjects.

## Introduction

Multiple system atrophy (MSA) is a sporadic, adult-onset, and progressive neurodegenerative disease. It has neuropathological features that lead to neuronal loss mainly in the nigrostriatal pathways, such as the basal ganglia, cerebellum, pons, inferior olivary nuclei, and spinal cord (Stefanova, 2009). MSA is clinically characterized as Parkinsonism, cerebellar ataxia, and autonomic dysfunction; the disease manifests as various combinations of corticospinal tract impairment and urinary symptoms (Gilman et al., 1999; Kaufman et al., 2013). It has been reported that the incidence of the disease is six patients per 1,000,000 persons per year (Stefanova, 2009). MSA is divided into three types: Parkinsonism (MSA-P), cerebellar dysfunction (MSA-C), and autonomic and urinary dysfunction (MSA-A) (Gilman et al., 1999; Wenning et al., 2004). There is evidence that a core protein, alpha-synuclein, is involved in the pathogenesis of MSA. This finding indicates that the disease may have a similar neuropathological characteristic to other α-synucleinopathies diseases, such as Parkinson’s disease (PD) (Kim et al., 2011; Yasuda et al., 2013; Saracchi et al., 2014). Although the etiology of MSA and PD are poorly understood, they share a possible pathogenesis and show similar clinical features. For instance, both are influenced by motor and non-motor dysfunctions such as rigidity and cognitive impairment.

Neuroinflammation has been described as an important participant in several neurodegenerative diseases including PD, Alzheimer’s disease (AD), and MSA (Gemma, 2010; Labandeira-Garcia et al., 2011; Rosano et al., 2012). It has been shown that the proinflammatory cytokine interleukin 18 (IL-18) is elevated in post-mortem AD patient brains, and it modulates the tau kinases, GSK3β, and Aβ-production, strongly implying that the chronic inflammation and oxidative stress are closely related to AD pathogenesis (Sutinen et al., 2014). Abnormal activation of microglia can cause damage to the brain–blood barrier (BBB) in the central nervous system (CNS), and the accumulation of activated microglia and proinflammatory cytokines may accelerate and amplify the neuroinflammation in neurodegenerative diseases such as PD, amyotrophic lateral sclerosis (ALS), and AD (Bachstetter and Van Eldik, 2010; Paredes et al., 2010; Bendotti et al., 2012; Gordon, 2013; Chaudhari et al., 2014; da Fonseca et al., 2014; Takeuchi and Suzumura, 2014). Several lines of evidence also indicated that neuroimmunomodulation may be a potential therapeutic strategy in delaying the neurodegenerative process (Gemma, 2010; Carret-Rebillat et al., 2015; Hardeland et al., 2015). One clinical study by Kaufman et al. demonstrated that IL-6 and TNF-α were significantly elevated in MSA patients when compared to healthy controls (Kaufman et al., 2013). C-reactive protein (CRP), a very sensitive and important inflammatory mediator, was been shown to be a valid predictor of new cardiovascular risks, type 2 diabetes, ischemic cerebrovascular disease, and PD (Fu et al., 2009; Song et al., 2009). Homocysteine (Hcy), a sulfur-containing amino acid, is produced by the interaction of cysteine and methionine and is closely related to cognitive impairment (Rodriguez-Oroz et al., 2009; Shimomura et al., 2011; Ray et al., 2013). Although the exact mechanisms are unclear, the evidence suggests that Hcy plays important roles in the modulation of *N*-methyl-d-aspartate (NMDA) receptors, oxidative stress, activation of caspases, DNA damage, and mitochondrial dysfunction (Lipton et al., 1997; Outinen et al., 1998; Kruman et al., 2000; Huang et al., 2001). Uric acid (UA) has gained popularity as a natural antioxidant and biomarker of oxidant stress. It is well documented that high levels of serum UA may have a neuroprotective role in some neurodegenerative diseases including PD and ALS (Paganoni et al., 2012; Cao et al., 2013). It is important to note that there is also evidence against this theory (Kim et al., 2011). Several studies, including ours, have demonstrated that some inflammation-related mediators in the peripheral circulation may be used as indicators for the severity of PD (Rodriguez-Oroz et al., 2009; Song et al., 2009; Pan et al., 2013). To the best of our knowledge, there is a paucity of studies that explore the combined effects of CRP, Hcy, and UA in evaluating patients with MSA.

This study was performed to investigate whether the inflammatory-related mediators, CRP, Hcy, and UA, are associated with the severity and prevalence of MSA. The primary aim of this study is to compare serum levels of CRP/Hcy/UA among MSA patients, PD patients, and normal healthy subjects. The secondary aim is to evaluate whether serum levels of CRP/Hcy/UA are associated with motor and non-motor dysfunctions and to identify their associations with different non-motor symptoms (NMS) domains in MSA. Lastly, our goal is to determine the diagnostic value of the serum levels of CRP/Hcy/UA in the patients with MSA.

## Materials and Methods

### Patients and ethics statement

From July 2011 to November 2014, a total of 47 patients with MSA (31 males and 16 females; mean age ± SD, 58.74 ± 10.18) (Table 1) who were admitted to the Department of Neurology of the Third Affiliated Hospital of Sun Yat-sen University and the Department of Neurology, Guangdong 999 Brain Hospital, Guangzhou, China, were enrolled in this cross-sectional study. The patients were identified according to the consensus criteria for the clinical diagnosis of MSA (Gilman et al., 1999). Additionally, 60 patients with PD (34 males and 26 females; mean age ± SD, 63.10 ± 10.62) (Table 1) were recruited based on the United Kingdom PD Society Brain Bank (UK-PDSBB) criteria (Hughes et al., 1992). A total of 50 healthy age-matched subjects (27 males and 23 females, mean age ± SD, 55.64 ± 10.82) (Table 1) were recruited from the outpatient setting as the control group. All the outpatients (control group) were recruited from the Medical Examination Center in the Third Affiliated Hospital of Sun Yat-sen University hospital. These subjects did not present with hypertension, cerebral ischemia, cardiovascular, diabetes, or renal dysfunction diabetes considering that presentation with any of the aforementioned diseases may influence the serum levels of UA, Hcy, and CRP. In addition, any prostate carcinoma-related mediators [prostate-specific antigen (PSA), carcinoembryonic antigen (CEA), or alpha-fetoprotein (AFP)] were initially measured in this study, and those who have high levels of those mediators were excluded from this study in order to eliminate the confounding factors.

**Table 1:** **Demographic, motor, and non-motor parameters**.

Clinical parameters	MSA			Control			PD		
	Mean (SD)	Min	Max	Mean (SD)	Min	Max	Mean (SD)	Min	Max
Gender (n)
Male n(%)	31 (66)			27 (54.0)			34 (56.7)		
Female n(%)	16 (34)			23 (46.0)			26 (43.3)		
Age (years)	58.74 (10.18)	23	79	55.64 (10.82)	38	76	63.10 (10.62)	41	80
UMSARS (total)	36.17 (10.49)	22	65						
UPDRS (I)	14.64 (4.43)	8	26						
UPDRS (II)	18.96 (6.35)	10	38						
UPDRS (IV)	2.81 (0.95)	1	5						
H&Y	3.21 (0.81)	2	5				2.18 (0.95)	1	5
MMSE	25.32 (3.42)	13	30				25.68 (3.71)	15	30
PDSS	122.60 (10.90)	93	143				113.58 (13.56)	60	130
NMSS (total)	72.43 (22.88)	26	123				60.18 (47.36)	5	188
Cardiovascular	8.55 (4.36)	0	16				2.97 (3.08)	0	13
Sleep/fatigue	12.26 (6.27)	1	26				13.35 (8.72)	0	36
Mood	13.49 (6.70)	4	30				13.02 (12.80)	0	54
Perceptual problem	1.96 (3.15)	0	10				1.38 (2.89)	0	12
Attention/memory	7.68 (4.40)	0	21				6.32 (6.04)	0	25
Gastrointestina	9.38 (4.73)	0	18				7.30 (5.62)	0	30
Urinar	9.34 (7.95)	0	24				7.03 (7.69)	0	32
Sexual function	3.45 (6.36)	0	24				3.20 (5.92)	0	21
Miscellaneous	7.13 (5.14)	0	18				4.55 (7.36)	0	36
Schwab & England	61.91 (21.02)	0	90				76.67 (12.31)	30	90
Webster	12.72 (3.35)	7	22				14.77 (11.13)	1	90

*SD, standard deviation; UMSARS, the unified Parkinson’s disease rating scale part; H&Y, the modified Hoehn and Yahr staging scale; MMSE, mini-mental state examination; PDSS, parkinson’s disease sleep scale (range of possible scores from 0 to 150), and NMSS, non-motor symptoms scale for Parkinson’s disease (range of possible scores from 0 to 360)*.

This study was approved by the local Ethics Committee of the Third Affiliated Hospital of Sun Yat-sen University and has been conducted according to the principle outlined in the Declaration of Helsinki of 1975 and the National Institute of Health Human Subjects Policies and Guidance released in January and December 23, 1999, respectively. All participants provided written consent for the investigation and the consent to measure the levels of serum CRP, Hcy, and UA and participate in the following standard assessments: the Unified MSA rating scale (UMSARS), PD sleep scale (PDSS), NMS scale (NMSS), the Schwab & England activities of daily living (ADL) scale, Webster scale, modified Hoehn and Yahr staging scale (H&Y), Mini-Mental State Examination (MMSE), and unified PD rating scale (UPDRS). All the assessments were conducted in a blinded manner.

### Study design

**Figure 1:**
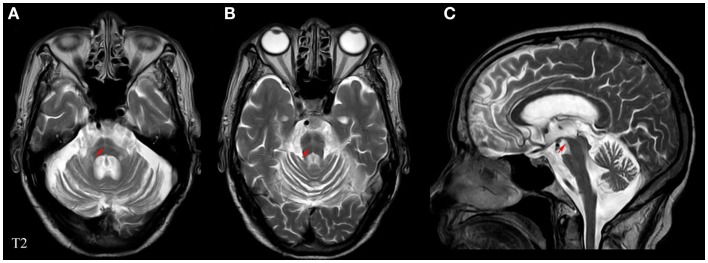
**Representative MRI images of a patient with MSA**. **(A)** Horizontal and **(B)** vertical hyperintensities in the pontine also known as “cross sign,” are shown in T2-weighted images. **(C)** Angle sign in the brainstem shown in a T2-weighted image. Red arrows indicate abnormalities pertaining to MSA.

Experienced neurologists were recruited to perform the evaluations and complete the neurological examinations for both the inpatients and the outpatients. All patients with MSA included in this study satisfied the criteria outlined in Wenning’s study (Wenning and Krismer, 2013); the patients with PD fulfilled the criteria of the UK-PDSBB (Hughes et al., 1992). The exclusion criteria were as follows: (1) all patients with disability due to neurological disorders other than MSA/PD, such as cerebral vascular disease, the related sequelae, or psychosis; (2) participants with diseases that could possibly influence NMS including pain syndromes, advanced diabetes, mellitus, malignancy, renal, hepatic failure or cardiovascular diseases, severe anemia, or any other acute or chronic debilitating or life-threatening disease/state; and (3) individuals who refused to participate in the study. All subjects completed the following battery of standardized assessment measures: the UMSARS was used to evaluate motor dysfunction and disease severity; the scales included a historical review of disease-related impairments (I, 12 items; the range is 0–48 points; a higher score represents more severe disability), detailed motor examination (II, 14 items; 0–56 points), and a global disability scale (IV) (Geser et al., 2005). The H&Y and the ADL scales divided patients into stages on the basis of clinical disability (Kaufman et al., 2013). The Webster scale (10 items, total score ranging from 0 to 30 points) was applied to assess the degree of motor disability. The degree of NMS in every patient was measured by the NMSS and PDSS (Zhang et al., 2011; Pan et al., 2013). Cognitive abilities were evaluated with the MMSE (Barone et al., 2009; Pfeiffer, 2009). The typical magnetic resonance imaging (MRI) for MSA patients is shown in Figure 1. All scales were available and validated for the Chinese population. The demographics and clinical data of the subjects are shown in Table 2.

**Table 2:** **Demographic, motor, and non-motor parameters in three MSA subtypes**.

Clinical parameters	MSA-P Mean (SD)	MSA-C Mean (SD)	MSA-A Mean (SD)
Gender (n)
Male (%)	5 (26.3)	9 (45.0)	2 (25.0)
Female (%)	14 (73.7)	11 (55.0)	6 (75.0)
Age (years)	58.53 (9.07)	57.90 (11.52)	61.38 (9.96)
UMSARS (total)	31.00 (5.96)	40.30 (11.81)	38.13 (11.32)
UPDRS (I)	12.53 (2.70)	16.30 (5.28)	15.50 (3.70)
UPDRS (II)	16.58 (4.19)	21.05 (6.66)	19.38 (8.42)
UPDRS (IV)	2.41 (0.69)	3.00 (1.08)	3.25 (0.89)
H&Y	2.74 (0.81)	3.45 (0.61)	3.75 (0.71)
MMSE	25.58 (4.00)	25.30 (3.16)	24.75 (2.82)
PDSS	123.11 (10.53)	120.80 (12.38)	125.88 (7.57)
NMSS (total)	78.68 (24.76)	66.90 (19.82)	71.38 (24.53)
Cardiovascular	7.11 (3.76)	8.40 (4.62)	12.38 (2.93)
Sleep/fatigue	14.11 (6.84)	11.30 (6.43)	10.25 (3.11)
Mood	14.47 (6.88)	13.35 (6.73)	11.50 (6.55)
Perceptual problem	2.42 (3.50)	2.25 (3.26)	0.13 (0.35)
Attention/memory	8.37 (4.57)	7.85 (4.16)	5.63 (4.47)
Gastrointestina	10.53 (4.64)	9.00 (4.72)	7.63 (4.90)
Urinar	9.53 (7.59)	7.30 (7.23)	14.00 (9.38)
Sexual function	3.68 (5.93)	3.20 (6.96)	3.50 (6.57)
Miscellaneous	11.21 (3.92)	3.75 (3.86)	5.88 (3.72)
Schwab & England	66.84 (20.29)	62.50 (14.82)	48.75 (31.37)
Webster	12.16 (2.43)	13.50 (3.99)	12.13 (3.52)
Daily dose of l-Dopa (mg)	305.26 (77.99)		
(MSA-P)			

*MSA-P, parkinsonism type; MSA-C, cerebellar dysfunction; MSA-A, autonomic and urinary dysfunction; SD, standard deviation; UMSARS, the unified Parkinson’s disease rating scale part; H&Y, the modified Hoehn and Yahr staging scale; MMSE, mini-mental state examination; PDSS, parkinson’s disease sleep scale (range of possible scores from 0 to 150) and NMSS, non-motor symptoms scale for Parkinson’s disease (range of possible scores from 0 to 360)*.

### Blood sampling measurement

Venous blood samples for CRP, Hcy, and UA measurements were obtained from all subjects in the study. A total of 5 mL of blood was taken from the patients, and all the measurements were replicated thrice. The serum was separated within 1 h by centrifugation at 3,000 rpm for 10 min. The separated sera were stored at –30°C until laboratory evaluation took place. The CRP level was measured by latex immunoturbidimetric assay, according to Ichihara’s study (Ichihara et al., 2002); the serum level of Hcy was determined using a solid-phase competitive chemiluminescent enzyme immunoassay (Rodriguez-Oroz et al., 2009). The UA level was measured by a Biochemical Analyzer 7180-ISE (Hitachi High-Technology Science Systems Corporation, Tokyo, Japan) using the UA assay kit via the URO-PAP method (Sinosource Biopharmaceutical Inc.) (Pan et al., 2013).

### Statistical analysis

All continuous variables, including age, UMSARS (UMSARS-I, -II, -IV and the sum of UMSARS-I, -II, and -IV), MMSE, NMSS, PDSS, Webster, CRP, Hcy, and UA, were presented as the mean ± SD; all categorical variables, including gender and subtype (C-type, P-type, or autonomic and urinary dysfunction), were presented as percentages. The total scores of age, UMSARS, MMSE, NMSS, PDSS, Webster, CRP, Hcy, and UA were counted by summing the single items. Statistical significance of differences between the groups was assessed using the non-parametric data Mann–Whitney U-test and Kruskal–Wallis test when the data were not normally distributed; Student’s *t*-test was used when the data were normally distributed. Tukey’s *post hoc* analysis was conducted to compare the differences of age/CRP/Hcy/UA among normal subjects, PD and MSA patients. The Spearman’s rank correlation coefficient (*r*_s_) was performed to evaluate correlations in different clinical parameters. A receiver operating characteristic (ROC) analysis was conducted to assess the performance of clinical biomarkers (Hcy, UA, and CRP) in the diagnostic accuracy for this disease. Additionally, the ROC curve for the combination of Hcy and UA was calculated to screen for a better prognostic tool using a logistic regression analysis. *p*-Values <0.05 were deemed statistically significant and SPSS 13.0 software (Chicago, IL, USA) was used for the statistical analyses.

## Result

### Patient characteristics

This cross-sectional study consisted of 47 MSA [31 males (66%) and 16 females (34%)], 60 PD patients [34 males (57%) and 26 (43%) females], and 50 healthy subjects [27 males (54%) and 23 females (46%)]. The mean age of MSA, PD and normal controls were 58.74 ± 10.18, 63.10 ± 10.62, and 55.64 ± 10.82, respectively (Table 1). There was no significant difference in the ages among the MSA patients and normal subjects (58.74 ± 10.18 *vs*. 55.64 ± 10.82, *p * = 0.319, Tukey’s test, Table 3), and MSA and PD patients (63.10 ± 10.62 *vs*. 58.74 ± 10.18, *p * = 0.089, Tukey’s test, Table 3). When the subjects were classified into three subtypes, the mean ages of MSA patients was 58.53 ± 9.07 (MSA-P), 57.90 ± 11.52 (MSA-C), and 61.38 ± 9.96 (MSA-A). There was no significant difference between ages among the three types (*p * = 0.949, Kruskal–Wallis test; data not shown). The demographic and clinical data for the subjects are shown in Table 2.

**Table 3:** **Comparison of age, CRP, Hcy, and UA among MSA, PD, and normal healthy subjects**.

Variable	MSA (mean ***±*** SD)	PD (mean ***±*** SD)	Control (mean ***±*** SD)	*χ^2^*	*p*-Value	Tukey’s	
						MSA/PD	MSA/Control
Age	58.74 ± 10.18	63.10 ± 10.62	55.64 ± 10.82	13.157	0.000***	0.089	0.319
CRP	1.70 ± 1.41	1.34 ± 1.09	1.38 ± 1.085	1.289	0.525	0.982	0.402
Hcy	13.28 ± 4.13	12.89 ± 5.70	10.34 ± 3.07	13.74	0.001**	0.897	0.005**
UA	315.28 ± 83.14	291.07 ± 71.21	357.49 ± 87.81	15.64	0.000***	0.272	0.029*

*p < 0.05, **p < 0.01, ***p < 0.001.

*Kruskal–Wallis test for the comparison among MSA, PD, and normal subjects, Tukey’s post hoc analysis for the comparison in MSA vs. PD or MSA vs. Control*.

### Comparisons of CRP/Hcy/UA between MSA/PD patients and healthy subjects

In this study, no significant difference of serum CRP levels among patients with MSA, PD patients, and healthy subjects (Table 3) was observed. However, significant differences of serum Hcy were found among PD, MSA, and the controls (**p  = 0.001, Kruskal–Wallis test, Table 3). The serum Hcy in MSA was higher than that in normal subjects (13.28 ± 4.13 *vs*. 10.34 ± 3.07, **p = 0.005, Tukey’s test, Table 3), but no significant difference in the Hcy levels between MSA and PD patients was found. Furthermore, the UA levels in patients with MSA were significantly lower than that in the healthy subjects (315.28 ± 83.14 *vs*. 357.49 ± 87.81, *p  = 0.029, Tukey’s test, Table 3). However, our data showed no significant differences in the CRP, Hcy, and UA levels among patients with MSA and PD (Table 3).

**Figure 2:**
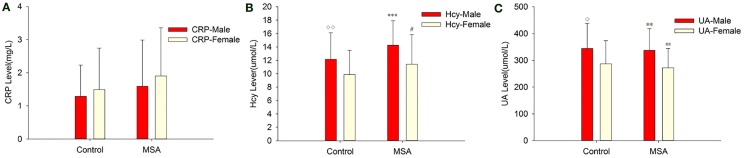
**Comparison of CRP, Hcy, and UA levels between MSA and control patients, according to gender**. **(A)** Comparison of CRP between control and MSA groups. No significant differences of CRP levels were found between MSA *vs*. control, MSA (male)*vs*. control (male), MSA (female)*vs*. control (female) and MSA (male) vs. MSA (female). **(B)** Comparison of Hcy between control and MSA groups. ***MSA (male) *vs*. control (male), *p* < 0.001; ^♢^MSA (male) *vs*. MSA (female), *p* = 0.023; ^##^MSA *vs*. control, *p * = 0.002. **(C)** Comparison of UA between control and MSA groups. **MSA (male) *vs*. control (male), *p* = 0.007; ^♢^MSA (male) *vs*. MSA (female), *p* = 0.009; ^##^MSA *vs*. control, *p * = 0.011.

When MSA patients and healthy subjects were divided into specific gender groups, the serum levels of Hcy in the male patients with MSA were higher than that of the normal male subjects (14.26 ± 3.67 *vs*. 10.77 ± 2.94, ***p  = 0.000, Student’s *t*-test, Table 4). Similarly, a significant difference was observed in plasma UA levels of MSA male patients and normal male subjects (337.45 ± 80.86 *vs*. 397.74 ± 84.49, **p = 0.007, Mann–Whitney *U*-test, Table 4). Interestingly, there was no significant difference between both Hcy and UA levels in female MSA patients and healthy female subjects. Additionally, the level of Hcy in male MSA patients was higher than that in the female patients (14.26 ± 3.67 *vs*. 11.40 ± 4.44, *p  = 0.023, Student’s *t*-test, Table 4); the serum UA levels in male patients were higher than those in female patients with MSA (337.45 ± 80.86 *vs*. 272.32 ± 71.67, **p = 0.009, Student’s *t*-test, Table 4). On the contrary, no significant differences in serum CRP levels were observed between the MSA patients and healthy subjects; there were also no significant differences in the serum CRP levels between male MSA patients and female MSA patients (Table 4; Figure 2).

**Table 4:** **Comparison of CRP, Hcy, and UA between normal subjects and MSA patients according to genders**.

Variable		MSA (mean ***±*** SD)	Control (mean ***±*** SD)	MSA vs. Control		MSA (Male) vs. (Female)	
				Value	*p*	Value	*p*
CRP	Male	1.59 ± 1.39	1.36 ± 0.96	−0.722	0.473^a^		
	Female	1.90 ± 1.46	1.41 ± 1.24	−1.128	0.267_b_	−0.842	0.400^a^
Hcy	Male	14.26 ± 3.67	10.77 ± 2.94	3.746	0.000***_b_		
	Female	11.40 ± 4.44	9.96 ± 3.18	−0.791	0.429_a_	2.349	0.023*^b^
UA	Male	337.45 ± 80.86	397.74 ± 84.49	−2.705	0.007**_a_		
	Female	272.32 ± 71.67	310.24 ± 66.45	1.698	0.098_b_	2.716	0.009**_b_

*p  < 0.05, **p < 0.01, ***p < 0.001.

*^a^Mann–Whitney U-test*.

*^b^Student’s *t*-test*.

### Correlations between CRP, Hcy, UA Levels and UMSARS, H&Y, MMSE, PDSS, NMSS, Schwab & England, and Webster

To evaluate the correlations between the severity of the disease and clinical variables, we conducted Spearman’s correlation analysis between the inflammatory-related mediators and various assessments. There were significant correlations between CRP and UMSARS-I (*r*_s_ = 0.326, *p  = 0.025, Table 5), CRP and UMSARS-IV (*r*_s_ = 0.419, **p  = 0.003, Table 5), CRP and UMSARS-total (*r*_s_ = 0.363, *p  = 0.012, Table 5), CRP and H&Y (*r*_s_ = 0.342, *p  = 0.019, Table 5), CRP and MMSE (*r*_s_ = −0.388, **p  = 0.007, Table 5), and CRP and Schwab & England (*r*_s_ = −0.370, *p  = 0.011, Table 5). Although no significant correlations were observed between CRP and UMSARS-II, CRP and PDSS, CRP and Webster, CRP and some of burdens of NMSS, there were significant correlations between CRP and NMS burdens of cardiovascular (*r*_s_ = 0.357, *p  = 0.014, Table 5) as well as perceptual problem (*r*_s_ = 0.336, *p  = 0.021, Table 5). Meanwhile, significant correlations were observed between plasma Hcy and UMSARS-IV (*r*_s_ = 0.327, *p  = 0.025, Table 5), Hcy and H&Y (*r*_s_ = 0.315, *p  = 0.031, Table 5), and Hcy and MMSE (*r*_s_ = −0.364, *p  = 0.012, Table 5) in MSA patients. In the nine domains of NMSS, we also found significant correlations between Hcy and NMS burden of cardiovascular in MSA patients (*r*_s_ = 0.354, *p  = 0.015, Table 5). There were however, no significant correlations between Hcy and UMSARS-I, Hcy and UMSARS-II, Hcy and UMSARS-total, Hcy and PDSS, Hcy and Schwab & England, Hcy and Webster, Hcy and other NMS burdens in MSA patients. No significant correlations were observed in UA and UMSARS (I/II/IV/total), UA and H&Y, UA and MMSE, UA and PDSS, UA and total/nine domains of NMSS, UA and Schwab & England, UA and Webster among MSA patients. Interestingly, our results also showed that 20 patients with MSA-P subtype were treated with L-dopa; however, L-dopa was not significantly correlated to the serum levels of Hcy, UA, and CRP (Table 5). This finding strongly suggests that L-dopa medication in MSA would not influence the validation of Hcy/UA/CRP assessment in MSA.

**Table 5:** **Spearman’s rank correlation coefficient (*r*_s_) and *p*-values between clinical variables and H&Y, MMSE, PDSS, NMSS (total/domain), Schwab & England, and Webster tests**.

Variable	CRP		Hcy		UA	
	*r*	*p*	*r*	*P*	*r*	*p*
UMSARS(total)	0.363	0.012*	0.193	0.195	0.067	0.653
UMSARS(I)	0.326	0.025*	0.096	0.521	0.169	0.256
UMSARS(II)	0.091	0.541	0.173	0.246	0.135	0.367
UMSARS(IV)	0.419	0.003**	0.327	0.025*	0.079	0.595
H&Y	0.342	0.019*	0.315	0.031*	0.169	0.255
MMSE	−0.388	0.007**	−0.364	0.012*	0.085	0.571
PDSS	−0.047	0.755	−0.031	0.836	−0.025	0.867
NMSS (total)	0.241	0.103	0.004	0.977	0.062	0.681
Cardiovascular	0.357	0.014*	0.354	0.015**	−0.205	0.168
Sleep/Fatigue	0.153	0.303	−0.230	0.120	−0.112	0.452
Mood	0.226	0.127	0.104	0.485	0.155	0.299
Perceptual problem	0.336	0.021*	−0.109	0.466	0.037	0.807
Attention/memory	0.089	0.554	−0.009	0.950	−0.158	0.289
Gastrointestina	0.114	0.446	−0.142	0.341	−0.069	0.645
Urinar	0.045	0.763	−0.020	0.894	0.016	0.915
Sexual function	0.219	0.140	0.029	0.846	0.177	0.233
Miscellaneous	−0.167	0.263	−0.139	0.352	−0.186	0.212
Schwab & England	−0.370	0.011*	−0.223	0.133	−0.104	0.487
Webster	0.231	0.118	0.230	0.119	0.070	0.642
Daily dose of L-Dopa (mg)	0.410	0.081	−0.023	0.897	−0.028	0.911

*p  < 0.05, **p  < 0.01.

**r*_s_, Spearman’s rank correlation coefficient; UMSARS, the unified Parkinson’s disease rating scale part; H&Y, the modified Hoehn and Yahr staging scale; MMSE, mini-mental state examination; PDSS, parkinson’s disease sleep scale and NMSS, non-motor symptoms scale for Parkinson’s disease*.

### The ROC Curve of CRP, Hcy, and UA in the Diagnosis of MSA

**Figure 3:**
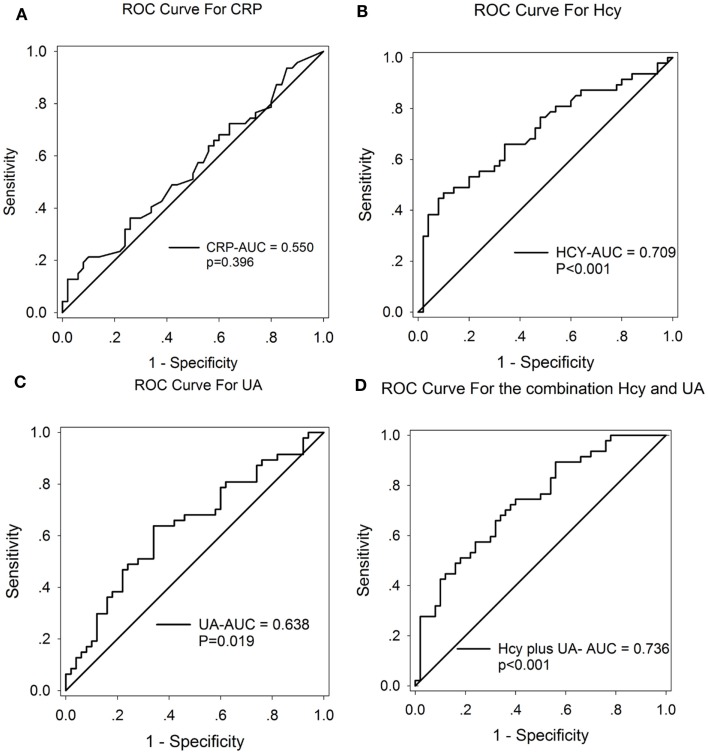
**ROC curves to evaluate the utility of serum levels of CRP, UA, and Hcy for the discrimination of MSA patients from healthy controls**. The AUC of ROC curves for **(A)** CRP, **(B)** Hcy, and **(C)** UA were 0.550 (95% CI: 0.434–0.666, *p * = 0.396), 0.709 (95% CI: 0.604–0.813, ***p  < 0.001), and 0.638 (95% CI: 0.527–0.749, *p  = 0.019), respectively. The AUC of **(D)** Hcy + UA was 0.736 (95% CI: 0.638–0.834, ***p  < 0.001).

A ROC curve was conducted to explore whether CRP, Hcy, and UA could provide credible discrimination between MSA patients and normal subjects. The ROC of Hcy analysis revealed that an area under the curve (AUC) value of 0.709 (95% CI: 0.604–0.813, ***p  < 0.001, Figure 3B) was observed; the cutoff was at 13.68 μmol/L, with a sensitivity of 53% and specificity of 90%. The AUC of UA was 0.638 (95% CI: 0.527–0.749, *p  = 0.019, Figure 3C); the cutoff was at 318.65 μmol/L, with a sensitivity of 64% and specificity of 66%. However, the AUC of CRP was 0.550 (95% CI: 0.434–0.666, *p * = 0.396, Figure 3A), indicating a non-significant difference. Furthermore, the AUC of the combination of Hcy and UA was 0.736 (95% CI: 0.638–0.834, ***p  < 0.001, Figure 3D), with a sensitivity of 74% and a specificity of 60% at the cutoff of 0.43 on the predicted risk algorithm, indicating that this combination variable was more robust than Hcy or UA alone in distinguishing MSA patients from healthy controls.

## Discussion

We found several interesting results in this study. First, we found a pronounced increase in the serum levels of Hcy and decrease in the levels of UA in MSA patients when compared to normal subjects. Second, the Hcy serum levels in male MSA patients were significantly higher than in male normal subjects; the UA serum levels were significantly lower than in male normal subjects. Third, the ROC curve analysis strongly indicated that the combination of Hcy and UA could significantly discriminate MSA patients from normal subjects and be applied as a new screening diagnosis instrument. The incorporated serum levels of Hcy and UA can lead to high yields and increase the diagnostic accuracy for differentiating MSA patients from healthy subjects. To the best of our knowledge, this is the first study exploring the association of serum Hcy/UA/CRP levels with the prevalence of MSA.

MSA is a neurodegenerative disease with an unknown etiology and pathogenesis. Therefore, it is important to explore the mechanisms of disease and specific biomarkers in evaluating the severity and progression of the disease with regard to motor and non-motor dysfunctions. There is evidence that inflammatory responses may be involved in the pathogenesis of neurodegenerative diseases such as AD, PD, ALS, and MSA (Wyss-Coray, 2002; Bachstetter and Van Eldik, 2010; Reese and Taglialatela, 2010; Sekiyama et al., 2012). There are similarities between PD and MSA including an abnormal accumulation of α-synuclein protein, a progressive disease course, and impaired motor and non-motor functions. According to previous studies, inflammatory mediators including CRP and Hcy may be used as biomarkers in evaluating the severity and progression of PD (Lindqvist et al., 2012; Shtilbans and Henchcliffe, 2012). Therefore, we examined whether those same inflammatory mediators may also be employed as viable and reliable biomarkers in assessing MSA. The associations between the inflammatory mediators and disease severity were explored in the current study. We also identified clinical variables that associated with MSA in different gender groups.

Our results showed no significant difference in serum CRP levels among MSA patients, PD patients, and healthy subjects. This finding is different from the changes of serum CRP in PD and AD; there are increased levels of CRP in PD and reduced levels in AD (Song et al., 2009; O’Bryant et al., 2010). Although serum CRP levels in MSA were higher than those in healthy subject controls, there was no statistically significant difference. One possible explanation may be that the elevation of CRP in the CNS is not high enough to affect its peripheral concentration in MSA patients (Chen et al., 2007). On the other hand, a study by Andican et al. showed that higher CRP levels only occur in the early stage of H&Y II in PD patients (Andican et al., 2012). In the current study, MSA patients recruited were far beyond the H&Y II stage. Serum levels of Hcy in these patients were significantly higher than those in healthy subjects, which is similar to other neurodegenerative diseases such as AD and PD (Zoccolella et al., 2010; Zhang et al., 2011; Ray et al., 2013; Kim and Lee, 2014). When compared to the healthy controls, the increased Hcy levels in MSA patients imply that Hcy contributes to the pathophysiological mechanism in the disease process; Hcy-mediated oxidative stress plays an important role in the pathogenesis of MSA. The precise mechanisms of Hcy’s neurotoxicity in MSA have not yet been elucidated, but we theorize that the higher levels of Hcy in MSA may damage endothelial cells. This can induce the damage of the BBB and lead to the generation of reactive oxygen species (ROS), which subsequently promotes the release of inflammatory cytokines and induces apoptosis (Harrison and Dexter, 2013; Kamat et al., 2013; Caldeira et al., 2014). It has been well documented that both CRP and Hcy have been reported to be associated with a high risk of prostate cancer, which usually occurs in elderly male persons. Higher serum Hcy can independently predict the risk of early biochemical recurrence and aggressiveness of prostate cancer (Stabler et al., 2011). Several lines of evidence also indicated that the elevated serum CRP levels were correlated to the high risk of prostate cancer and its assessment (Toriola et al., 2013; Thurner et al., 2015). Therefore, in the diagnosis and evaluation of MSA, those with high serum levels of PSA, CEA, or AFP were unsuitable for this study.

Interestingly, we observed no significant difference in the serum Hcy levels between MSA and PD patients. This finding supports the point that there may be similarities between the neuropathogenesis of MSA and the PD. Previous studies have demonstrated that serum levels of UA are closely correlated to the severity of dopaminergic impairment in PD (Moccia et al., 2014). As a natural anti-oxidative stress substance, high levels of UA may protect neurons from apoptosis in MSA patients (Chen et al., 2013; Schwarzschild et al., 2014). Similar results have also been observed in PD patients (Pan et al., 2013; Simon et al., 2014). These findings strongly suggest that lower levels of UA may contribute to the severity of PD and MSA due to the reduced anti-oxidative protective effects; therefore, UA may be a possible modifier in MSA (Kim et al., 2011).

After dividing the subjects according to genders, we saw interesting observations when comparing MSA patients and healthy subjects (Table 4; Figure 2). Serum Hcy and UA levels in male MSA patients were significantly higher than female MSA patients. One possible reason is that males express more negative risk factors for disease such as smoking, drinking, and unhealthy diets than females (Baccarelli et al., 2007; Sobczak et al., 2007; Lee et al., 2010). These factors may upregulate baseline Hcy levels in MSA male patients, which were not considered in this study. We also found lower serum UA levels in male MSA patients compared to healthy male subjects; this difference was not significant in the females. This finding suggests that UA may be more useful in evaluating MSA in men. The bio-physiological associations between gender-specific hormones and UA may at least partly be attributable to a higher occurrence of MSA in men (Cao et al., 2013).

Hcy demonstrated a strong positive correlation with H&Y and NMSS (cardiovascular domain) and a negative correlation with MMSE in MSA patients. This finding strongly suggests that Hcy significantly influences the motor and non-motor dysfunctions of MSA patients. One of the purposes of this study is to explore if the inflammation biomarkers could be used to assess MSA in different NMS such as the cardiovascular domain. We noticed a significant correlation between inflammation biomarkers with the cardiovascular domain among MSA patients, strongly indicating that Hcy could be a relatively precise biomarker for NMS when evaluating MSA patients. Although Hcy is known for being a high risk factor for cardiovascular disease, our current study of 47 MSA patients only showed that 2 patients presented with mild T-wave depression in the ECG examination (data not shown). Moreover, none of the MSA patients displayed abnormal serum levels of creatine kinase (CK), CK-isoenzyme (CK-MB), myocardial enzymes (MGB), lactate dehydrogenase (LDH), and LDH isoenzyme (data not shown), which are markers for cardiovascular disease. This emphasizes the fact MSA patients do not necessarily present with cardiovascular diseases, and that Hcy would be a reliable biomarker for the cardiovascular aspect in MSA. Serum Hcy levels also have a relationship with neuropsychiatric diseases such as depression (Bjelland et al., 2003). Several reports have suggested a close relationship between Hcy and hippocampus atrophy in alcoholic patients, suggesting that Hcy may have an effect on cognitive function (Kurth et al., 2004; Shimomura et al., 2011). Our previous studies indicate that cognitive function is closely associated with neurotransmitter receptors, especially NMDA receptors in the hippocampus, caudate putamen, nucleus accumbens, cingulate cortex, and other areas in the brain (Xu et al., 2012; Wang et al., 2014). In fact, Hcy could constantly activate NMDA receptors to result in neurotoxicity (Boldyrev et al., 2013). Taken together, these studies indicate that Hcy may have an influence on the decreased cognition in MSA patients via a change in hippocampus volume and the modulation of NMDA receptors in the hippocampus. Interestingly, our results showed that serum UA had no significant correlation with the severity of both motor and non-motor dysfunctions in MSA patients. This finding implies that the relationship of UA with the development and exacerbation of MSA may not be clear. Based on this finding, we hypothesize that UA may have an important function in the pathogenesis (but not in the progression) of MSA. The results of our study strongly indicate that as common inflammatory mediators, Hcy, but not UA, may contribute to the prevalence and exacerbation of the disease.

Although screening tools such as MRI maybe useful in detecting late-stage MSA, it lacks the sensitivity in detecting early stage MSA. There remains a need to use more sensitive tools such as serum Hcy and UA measurements when screening for early stage MSA. Our ROC data yielded an AUC of 0.709 for Hcy and 0.638 for UA. These findings indicate an acceptable sensitivity and specificity of Hcy and UA in the potential discrimination of MSA. Notably, Hcy displays more reliable discrimination when compared to UA (Figures 3B,C). Moreover, one of the most notable findings in the current study showed that the combination of Hcy and UA exhibited a better discriminatory ability, with an AUC of 0.736, compared with Hcy or UA alone. The reliability and potential utility of combined Hcy and UA as diagnostic biomarkers in plasma for screening MSA is reflected in the ROC analysis. Our findings have important clinical relevance. With the use of the serum Hcy and UA as a screening tool, clinicians will potentially be able to detect MSA and easily screen early stage MSA patients. However, we must acknowledge that there may be a significant overlap between the serum levels of Hcy and UA in MSA and normal controls. This finding suggests that either Hcy or UA alone is not a reliable diagnostic predictor.

There are several limitations to our study, which should not be ignored: (1) a small number of participants (47 MSA, 60 PD, and 50 normal subjects) were recruited; (2) most patients with MSA are at early stages of the disease, with a low median stage of the UMSARS and a relatively high MMSE score (25.32 ± 3.42); (3) genetic factors such as the MTHFR genotype and folate or vitamin B12 administration were not considered in this study; (4) to validate and complete the questionnaire, we only chose MSA patients with sufficient cognitive ability; this significantly narrowed the population in the study. The above criteria (1–4) in the population chosen may have resulted in a bias for Hcy and UA levels in MSA patients. Therefore, it is necessary to conduct large population studies in the future. Furthermore, different stages of MSA patients in both UMSARS-IV and MMSE scores should be included to compensate for the limitations in the current study.

In summary, the current study supports the notion that inflammation contributes to the pathogenesis of MSA. Of the biomarkers studied (CRP, Hcy, and UA), Hcy was the most suitable and reliable inflammatory mediator in evaluating the severity of MSA. Based on this study, it is reasonable to speculate that high levels of serum Hcy may negatively impact MSA patients through various mechanisms, including generation of oxidative stress and persistent activation of NMDA receptors in the brain. The inflammatory biomarker Hcy, which distinguishes MSA patients from normal subjects, is important as it provides a testable hypothesis related to the pathophysiology of MSA. Furthermore, high levels of serum Hcy may predispose patients to the progressive stages in both motor and non-motor dysfunctions. The ROC curve, in combination with serum Hcy and UA, would be valuable for the early diagnosis of MSA; this method could be used to increase the diagnostic accuracy for differentiating MSA subjects from healthy subjects. Our findings support the use of serum Hcy and UA to diagnose MSA, but neuropathological correlation would be required to confirm this. To our best knowledge, this is the first study to consider the combination of serum Hcy and UA levels in diagnosing and assessing the severity of MSA. Further studies are needed to determine whether serum Hcy and UA abnormalities are indeed reliable parameters in discriminating MSA from PD, particularly in the early stages of MSA.

## Author Contributions

DC, XW, JZ, XL, and QW conceived and designed the experiments, DC, KL, XW, and QW performed the experiments, DC, RW, XX, XL, and QW analyzed the data, BT and ZW contributed reagents/materials/analysis tools, and BW, KJ, and QW wrote the paper.

## Conflict of Interest Statement

The authors declare that the research was conducted in the absence of any commercial or financial relationships that could be construed as a potential conflict of interest.

## Abbreviations

AD, Alzheimer’s disease; ADL, the Schwab & England activities of daily living scale; ALS, amyotrophic lateral sclerosis; AUC, area under the curve; BBB, blood–brain barrier; CRP, C-reactive protein; CNS, central nervous system; Hcy, homocysteine; H&Y, modified Hoehn and Yahr staging scale; MMSE, mini-mental state examination; MSA, multiple system atrophy; MSA-A, multiple system atrophy-autonomic and urinary dysfunction; MSA-C, multiple system atrophy-cerebellar dysfunction; MSA-P, multiple system atrophy-parkinsonism; NMDA, *N*-methyl-d-aspartate; NMS, non-motor symptoms; PD, idiopathic Parkinson’s disease; PDSS, Parkinson’s disease sleep scale; ROC, receiver operating characteristic; ROS, reactive oxygen species; *r*_s_, the Spearman’s rank correlation coefficient; SD, standard deviation; UA, uric acid; UK-PDSBB, the United Kingdom PD Society Brain Bank; UMSARS, unified multiple system atrophy rating scale.
